# Longitudinal changes in attention bias to infant crying in primiparous mothers

**DOI:** 10.3389/fnbeh.2023.1192275

**Published:** 2023-09-22

**Authors:** Daiki Hiraoka, Kai Makita, Nobuko Sakakibara, Shigemi Morioka, Makoto Orisaka, Yoshio Yoshida, Akemi Tomoda

**Affiliations:** ^1^Research Center for Child Mental Development, University of Fukui, Fukui, Japan; ^2^The Japan Society for the Promotion of Science, Tokyo, Japan; ^3^Department of Pediatrics, Fukui Aiiku Hospital, Fukui, Japan; ^4^Department of Pediatrics, Japanese Red Cross Kyoto Daini Hospital, Kyoto, Japan; ^5^Faculty of Medical Sciences, Department of Obstetrics and Gynecology, University of Fukui, Fukui, Japan; ^6^Department of Child and Adolescent Psychological Medicine, University of Fukui Hospital, Matsuoka-Shimoaizuki, Fukui, Japan; ^7^Division of Developmental Higher Brain Functions, United Graduate School of Child Development, Osaka University, Kanazawa University, Hamamatsu University School of Medicine, Chiba University, and University of Fukui, Osaka, Japan

**Keywords:** infant crying, attentional bias, postpartum change, Stroop task, maternal cognition

## Abstract

**Introduction:**

Infant stimuli attract caregiver attention and motivate parenting behavior. Studies have confirmed the existence of attentional bias toward infant face stimuli; however, relatively little is known about whether attentional bias exists for infant cry stimuli, which are as important as faces in child-rearing situations. Furthermore, scarce longitudinal evidence exists on how attentional bias toward infant crying changes through the postpartum period.

**Methods:**

In the present study, we conducted an experiment to assess bias toward infant crying at two postpartum time points: at Time 1 (Mean = 75.24 days), 45 first-time mothers participated and at Time 2 (Mean = 274.33 days), 30 mothers participated. At both time points, the mothers participated in a Stroop task with infant crying and white noise as the stimuli. They were instructed to answer the color out loud as quickly and accurately as possible, while ignoring the sound. Four types of audio stimuli were used in this task (the cry of the mother’s own infant, the cry of an unfamiliar infant, white noise matched to the cry of the mother’s own infant, and white noise matched to the cry of an unfamiliar infant), one of which was presented randomly before each trial. Response time and the correct response rate for each condition were the dependent variables.

**Results:**

For response time, the main effect of familiarity was significant, with longer response times when the participant’s infant’s cry was presented. In addition, response times were lower at Time 2 than at Time 1 in some conditions in which crying was presented.

**Discussion:**

The results suggest that mothers may be less disturbed by infant crying as they gain more experience. Elucidating the characteristics of postpartum mothers’ changes in cognitive performance related to infants’ cries would be useful in fundamental and applied research to understand the process of parents’ adaptation to parenting.

## Introduction

Reflexive and biased attention to ecologically important stimuli leads to efficient information processing and rapid intuitive responses, which increases individual and species survivability. Infants’ signals are different from those of adults, and they function to attract caregivers’ attention and motivate caregiving behavior. Adults rate faces with more infantile features as cuter and are more eager to care for them (Glocker et al., 2009; Endendijk et al., 2018). To examine attentional bias toward infant face stimuli, cognitive tasks (e.g., the dot-probe task and go/no-go task) are used to detect changes in response time to the presentation of infant faces. Several studies have confirmed that response times are shorter when an infant’s face is presented compared with when an adult’s face is presented; that is, adults’ attention is allocated more to infants’ faces [for a review, see Lucion et al. (2017)].

Caregivers’ attentional bias toward infant face stimuli is modulated by their mental health. Perinatal depression is a major mental health risk in the perinatal period that interferes with sensitive parenting of infants in the postpartum period. Depressive symptoms distort cognition, and biased attention to negative stimuli is associated with the onset and persistence of depressive symptoms (Beck, 2008; Disner et al., 2011). However, previous research examining the relationship between depressive symptoms during pregnancy and bias toward infant distress has shown that women with depressive symptoms have lower attentional bias toward infant distress than do women without depressive symptoms (Pearson et al., 2010). It has also been shown that lower attentional bias leads to difficulties in bonding with infants (Pearson et al., 2011). These findings suggest that bias toward infant stimuli has an adaptive aspect as well as that the inability to show bias may lead to mental health issues and mother–child relationship difficulties in the perinatal period.

Compared with the extensive research using infant face stimuli, few studies have used infant cry stimuli. Infants’ cries, like their faces, have different characteristics from those of adults, attracting caregivers’ attention and motivating parenting behavior. In particular, crying is a signal by which infants communicate their distress and physiological needs to their caregivers immediately after birth; it is important for their survival and attachment formation with their caregivers (Soltis, 2004). Studies have found that presenting infant crying during a cognitive task decreases task performance, suggesting the existence of attentional bias (Chang and Thompson, 2011; Hechler et al., 2015; Dudek et al., 2016). Dudek et al. (2016) presented infant vocalizations (crying and laughter) during or before a Stroop task trial and found that listening to an infant crying shortened response time compared with listening to laughter.

Pregnancy and childbirth alter the structure of the mother’s brain and these changes are believed to be adaptive to parenting behavior (Kim et al., 2010; Hoekzema et al., 2017, 2020). In animal models, females who have never given birth or raised offspring typically show indifference or avoidance toward unfamiliar pups. However, their responses shift to reward pup-related stimuli due to increased hypothalamic activity late in pregnancy and after delivery (Barrett and Fleming, 2011). In humans, parents show higher attentional bias toward infant face stimuli than do non-parents (Thompson-Booth et al., 2014a,b; Oliveira et al., 2017); the bias toward infant stimuli exhibited by parents may thus have an adaptive aspect that motivates sensitive responses to infant stimuli and prompt caregiving.

However, while it has recently been suggested that postpartum mothers’ responses to crying are not fixed and vary with their parenting experience, empirical findings on longitudinal changes in postpartum responsiveness are scarce. Bilgin and Wolke (2020) found that parents are initially less likely to leave their crying babies unattended immediately after birth. However, the frequency of this non-responsive behavior tends to increase gradually over time. It has also been shown that over the postpartum period, there is a gradual increase in orientation toward adults who are listening to the crying and not the crying baby (Hiraoka et al., 2021). Further, while previous studies have used subjective ratings of behavior and beliefs about crying, attentional bias is latent, less influenced by intentions and social desirability, and associated with the risk of psychiatric disorders. Therefore, examining changes in attentional bias and its association with the risk of psychiatric disorders is of great fundamental and clinical significance.

The primary objective of this research is to expand our understanding of cognitive functions in parenting behavior. Nevertheless, the application of our findings is not confined to parenting research alone. It has potential implications for a broader range of psychological domains, particularly those concerning emotions and sociality. Empathy, for instance, is a vital psychological capability that motivates not only parenting behavior but also our understanding of and assistance to others (Swain, 2011; Decety et al., 2012). Longstanding discussions suggest that empathy does not arise spontaneously; instead, it necessitates cognitive top-down control (Rameson et al., 2012; Spunt and Lieberman, 2013). This hypothesis also applies to the empathy that a mother feels toward her infant (Hiraoka and Nomura, 2016). An interaction between lower-level emotional arousal and higher-level control is deemed necessary for parenting (Kim et al., 2016; Swain and Ho, 2017), and a similar dual-pathway model has been proposed for empathy (Yu and Chou, 2018). However, to deepen our understanding of the specific mechanisms and applications of this model, further research is warranted. This study, which investigates changes in cognitive control in response to emotional stimuli, anticipates contributing empirical evidence to the broader context of empathy, serving as an important step toward the substantiation of the theoretical model.

### Aim and hypotheses

The main purpose of this study was to examine whether there is a bias in mothers’ attention toward infant crying and whether this bias is altered by parenting experience. For this purpose, first-time postpartum mothers were recruited to complete a Stroop task in which infants’ cries (their own and unfamiliar infants) were employed as distractors. We then obtained several subjective ratings of each cry, including caregiving intentions. In addition, postpartum depressive symptoms were measured using the Edinburgh Postnatal Depression Scale (EPDS) to examine how attentional bias toward infant crying is related to subjective ratings of crying and postpartum depressive symptoms. We then administered the same task approximately 6 months later to examine the changes in attentional bias within individuals. We hypothesized the following:

*Hypothesis* 1: Mothers exhibit attentional bias toward infant crying. Similar to bias toward infant face stimuli, bias toward infant crying is positively associated with caregiving intentions and negatively associated with postpartum depressive symptoms.

*Hypothesis* 2: Attentional bias toward infant crying changes with parenting experience. More specifically, in the immediate postpartum period, mothers are more sensitive to crying and allocate more attention to it than control sound.

## Materials and methods

### Participants

The sample participants were part of a longitudinal study of postpartum changes in new mothers conducted at the University of Fukui. The mothers participated in the experiment twice: at 2 months postpartum (Time 1) and 8 months postpartum (Time 2). Time 1 was an average of 75.24 days (*SD* = 12.85) postpartum, while Time 2 was an average of 274.33 days (*SD* = 15.23). An infant’s crying time and frequency peak approximately 1 month after birth and gradually decline thereafter (Barr, 1990; Vermillet et al., 2022). A longer crying duration and increased frequency could lead to heightened parenting stress and an increased risk of abuse (Lee et al., 2007; Fujiwara et al., 2011). Furthermore, while mothers gradually increase parent-oriented beliefs toward their infant’s cry, no significant change is observed between 0–2 months and 4–6 months in the postpartum period (Hiraoka et al., 2021). However, a significant change is detected during the four to 5 months after the third month postpartum. Based on these findings, we hypothesized that changes indicative of adaptation to crying occur between two to 3 months and 8 months (i.e., after the peak of crying). This informed the selection of our measurement time points for this study.

Forty-five mothers (mean age = 30.69 years, *SD* = 4.04) participated (October 2020 to September 2021) at Time 1. The participants were recruited from local maternity hospitals and university hospitals using a poster from July 2020 to March 2021. The inclusion criteria specified primiparous biological mothers with no self-reported hearing impairments. Of the participants who participated at Time 1, 30 took part in the Time 2 experiment conducted from March 2021 to March 2022. This study was approved by the university of Fukui’s Research Ethics Committee. Participation was voluntary and all participants provided written informed consent.

### Procedure

#### Stroop task

To examine attentional bias toward infant crying, a paradigm was created using the Stroop task adopted in a previous study (Dudek et al., 2016). Dudek et al. (2016) carried out two versions of a Stroop task: one in which infant crying or laughing sounds were presented immediately before each trial and another where these sounds were presented simultaneously with the trial. They consistently demonstrated that the valence of the vocalizations significantly influenced response times in both versions. Interestingly, the disruption effect of the sound valence was more potent when the sounds were presented just before the trials (*η^2^* = 0.53 vs. *η^2^* = 0.29).

This approach of presenting the sounds before each trial guided the design of this study, but it was modified and used to fit our purpose. Each trial began with the presentation of a fixation cross for 2,000 ms, followed immediately by the infant crying sound (or noise sound) for 2,000 ms paired with a fixation cross. Audio stimuli were presented via headphones (ATH-M30x, Audio-Technica, Japan). This was succeeded by the color words (“green,” “blue,” “red,” and “yellow”) or a control text (“xxx”) that appeared in red, blue, yellow, or green after 200 ms. Color words appeared as (1) congruent (e.g., the word “RED” printed in red; congruent condition), (2) incongruent (e.g., the word “RED” printed in blue; incongruent condition), and (3) control (i.e., “xxx” was presented in each of the four colors; control condition). Each of these three Stroop conditions comprised 48 trials, leading to a total of 144 trials. All the participants completed a four-trial practice run before the actual task. The participants’ responses were recorded as audio files, and the content of their speech and response latency were recorded for each trial. The response content was extracted using SpeechRecognition and the Python speech recognition library. Response time was determined using Chronset (Roux et al., 2017), as the time taken between the presentation of the word and first utterance. Response times shorter and longer than the range of the 2.5 × median were considered to be an absolute deviation from the median and excluded as outliers (Leys et al., 2013). Of the 10,656 trials in all Time 1 and Time 2 experiments for all participants combined, 573 trials (5.38%) were excluded as outliers. As an index of attentional bias toward infant crying, the difference in the mean response times in the crying and white noise trials was computed for each participant (sound type condition). Similarly, the difference in the mean response times in the own and unfamiliar infant trials was calculated for each participant as a measure of attentional bias toward their own infant’s cry (familiarity condition).

Mothers recorded their infants’ crying sounds at home a day before the experiment for use in the task. They were directed to capture spontaneous crying moments of their infants, such as before feeding or falling asleep, for a duration ranging from 30 s to 1 min using their smartphones. In addition to the recordings, participants provided contextual details and reasons related to the crying episode. These details have been tabulated and are available in Supplementary Table S1. Upon receipt of the recordings, our research team conducted a thorough review of each file. Our primary focus was on the initial 2,000 ms of the audio data, ensuring it was free of external interferences like other voices, background noises, or extended silences. Given the potential variability in the intensity of crying across different recordings, we sought to standardize the amplitude. This was achieved using the “Normalization” function in Audacity, a freely available open-source audio software, which can be downloaded.^1^ While amplitude was standardized, we made a deliberate decision not to modify the frequency of the crying sounds. This is because frequency is a unique acoustic characteristic of each cry. Retaining this natural variation is essential, as it potentially influences caregivers’ perceptions and reactions to the crying.

Regarding the unfamiliar infant cries used as stimuli in this study, we prepared a cry from a infant who matched the age of the participants’ own infants, specifically at two time points: 51 and 247 days postpartum. The cry was sourced from an infant whose parent was not part of the study. The processing of the unfamiliar cry underwent the same normalization procedure as the cries from the infants of participating mothers. Participants were informed only of the inclusion of “crying sounds and white noise” without specific mention of the origin or familiarity of these cries. Although they knew their infant’s cries would be part of the experiment due to the pre-experiment recording instructions, they were likely unaware of the inclusion of unfamiliar infant cries.

White noises corresponding to the own and unfamiliar infant’s cries were created as the control stimuli for each participant. The sound envelope and amplitude of the white noise stimuli were matched to the cries of their own and unfamiliar infant using the Audacity function. Therefore, four types of audio stimuli were used in this task (the cry of the mother’s own infant, the cry of an unfamiliar infant, white noise matched to the cry of the mother’s own infant, and white noise matched to the cry of an unfamiliar infant). One of the four types of sounds was presented in random order in each trial.

After completing the Stroop task, the participants were asked to rate each sound’s valence, arousal, urgency, health, and caregiving intentions on a five-point Likert scale (soothing – aroused, unpleased – pleased, non-urgent – urgent, healthy – sick). Additionally, they were asked to rate their feelings of wanting to pick up a crying baby (caregiving intention) on a five-point scale. The items we used were based on those employed in several previous studies that assessed perceptions of infant crying in a laboratory setting (Zeskind and Lester, 1978; Zeifman, 2004; Out et al., 2010; Lin and McFatter, 2012).

#### Questionnaire

After completing the cognitive tasks, the participants were asked to fill out a questionnaire to provide their demographic information and postpartum depressive symptoms as soon as possible at home. Postpartum depressive symptoms were evaluated using the EPDS (Cox et al., 1987), which has been validated and is widely used in clinical and research settings. It is a self-report questionnaire comprising 10 items assessing depressive symptoms that have occurred within the last 7 days. Each item is rated on a four-point scale and the total score is computed by averaging the item scores. In this study, the EPDS Cronbach’s alpha at Time 1 was 0.76 and at Time2 was 0.87, indicating sufficient internal consistency.

### Statistical analysis

For response time, all incorrect response trials (83 trials, representing 0.779% of all trials) were excluded from the analysis. We utilized a linear mixed-effects model (LMM) to analyze both response time and the error response rate. The model integrated fixed effects for the Stroop condition (congruent, incongruent, and control), sound type (crying and white noise), familiarity (own and unfamiliar infant), and time point (Time 1 and Time 2). Beyond a random intercept for participants, we incorporated interaction intercepts (id: Sound, id: Condition, id: Familiarity, and id: Time) to model random effects for all combinations of the levels of each subject and factor.

Our analytical approach proceeded as follows: Initially, we estimated a model that included only random effects (Model 1). Subsequently, Model 2 was constructed, incorporating control variables like age of participants at Time 1, age of the child at Time 1, and the interval in days between Time 1 and Time 2. Model 3 was defined by experimental factors, specifically Condition, Sound, Familiarity, and Time. Model 4 comprised interactions of interest, integrating Time, whereas Model 5 added secondary interactions of interest involving Time. The fit of these models was compared using likelihood-ratio tests (Tables 1, 2).

**Table 1:** Model fit indices for the response time.

Model	Specification	AIC	BIC	LRT		
				Statistic	df	*p* value
Model 1	Random Effects Only	7,444.25	7,476.30			
Model 2	Random Effects + Control Vars	7,438.20	7,483.99	2.82	3	0.42
Model 3	Random Effects + Control Vars + Main Effects	7,332.59	7,401.28	91.06	5	< 0.01
Model 4	Random Effects + Control Vars + Main Effects +1st Interactions	7,310.61	7,397.61	9.79	4	0.04
Model 5	Random Effects + Control Vars + Main Effects +1st Interactions +2nd Interactions	7,261.45	7,394.25	11.11	10	0.35

LRT, likelihood ratio test.

**Table 2:** Model fit indices for the error rate.

Model	Specification	AIC	BIC	LRT		
				Statistic	df	*p* value
Model 1	Random Effects Only	−3,322.48	−3,299.58			
Model 2	Random Effects + Control Vars	−3,278.57	−3,241.94	3.47	3	0.32
Model 3	Random Effects + Control Vars + Main Effects	−3,232.71	−3,173.18	13.8	5	0.02
Model 4	Random Effects + Control Vars + Main Effects +1st Interactions	−3,187.58	−3,109.73	1.65	4	0.80
Model 5	Random Effects + Control Vars + Main Effects +1st Interactions +2nd Interactions	−3,086.18	−2,962.54	7.93	10	0.64

LRT, likelihood ratio test.

After model selection, we delved into the fixed effects of the final model to test our hypotheses. Additionally, we computed the marginal and conditional *R^2^* statistics. For instances where significant main effect(s) and interaction(s) were observed in the final model, we estimated the marginal means for each condition and assessed the differences in scores across these conditions. Before the main analysis, data from participants who did not participate in Time 2 were excluded.

Subsequently, a Pearson correlation analysis was executed at each time point to investigate the relationships among participants’ attentional bias toward infant crying, subjective ratings of crying, and postpartum depressive symptoms. The bias score for crying was characterized as the difference in the mean response times between the sound type conditions. Similarly, the bias score for the mother’s own infant’s cry was denoted as the difference in the mean response times in the familiarity conditions. Statistical significance was set at *p* < 0.05. The LMM and subsequent tests were conducted on R (version 4.2.1) using the lmertest (Kuznetsova et al., 2017), emmeans (Lenth, 2022), and performance (Lüdecke et al., 2021) packages.

## Results

### Response time

**Figure 1:**
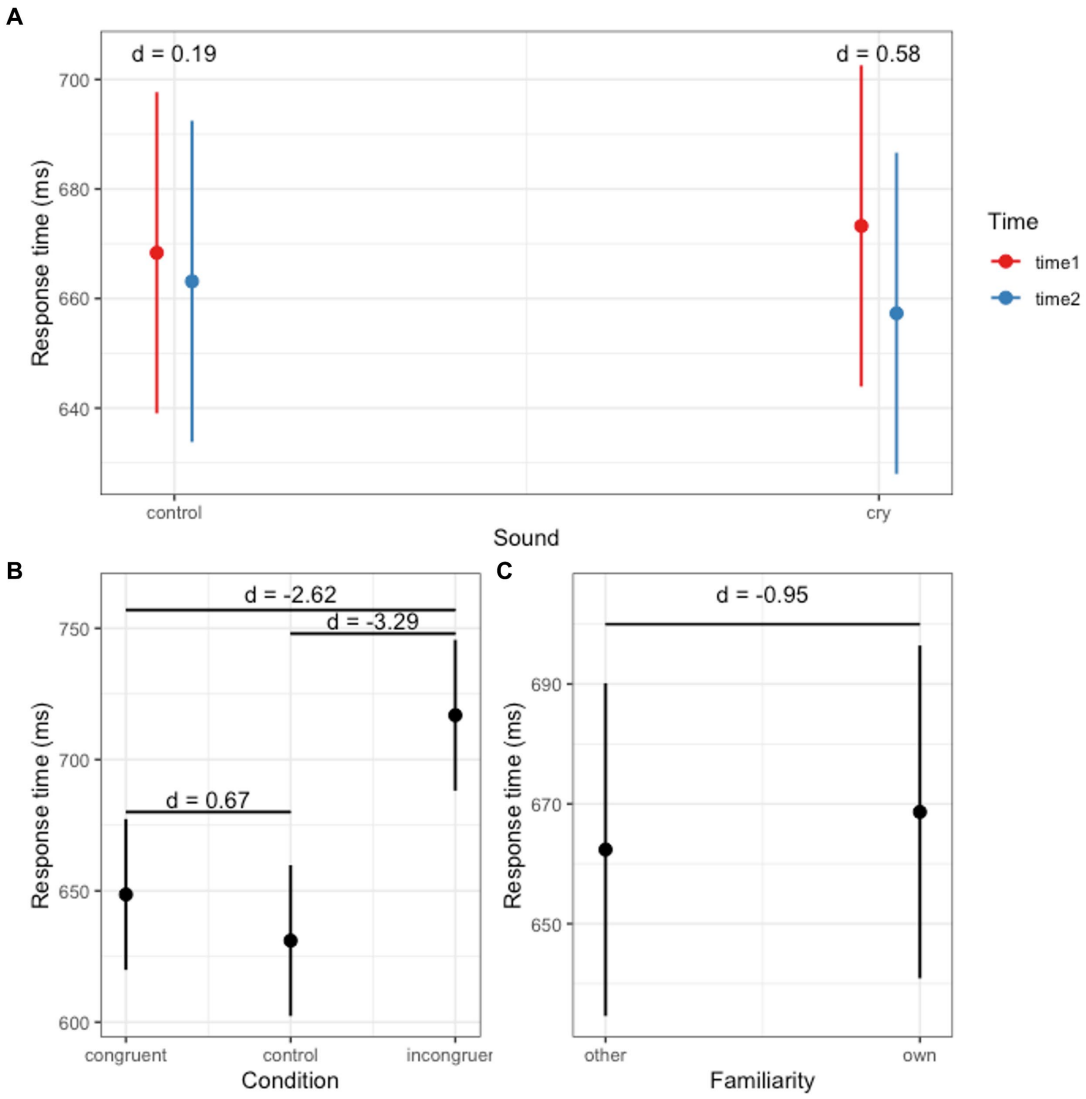
Response times across conditions. **(A)** Interaction effect between sound and time. **(B)** Main effect of Stroop condition. **(C)** Main effect of familiarity.

Table 1 presents the results for model fit and the likelihood-ratio tests. Model 4, which incorporated first-order interaction terms, was selected as the final model. Based on the outcomes from the final model (as shown in Table 3), we identified a significant main effect for both the Stroop condition and familiarity (Figures 1B,C). Additionally, a significant interaction between Time and Sound was observed (Figure 1A).

**Table 3:** Linear mixed model results for the response time.

Fixed effects				
	F	Df	Df.res	*p* value
Age	0.71	1	26	0.41
Child age	2.23	1	26	0.15
Interval	0.13	1	26	0.73
Condition	79.54	2	79.81	0
Sound	2.11	1	102.46	0.15
Familiarity	4.13	1	101.17	0.04
Time	0.83	1	45.26	0.37
Condition:Time	2.24	2	536	0.11
Sound:Time	5.27	1	536	0.02
Familiarity:Time	0.06	1	536	0.8
Random effects				
	Variance	SD		
id:Condition	579.62	24.08		
id:Time	1,222.27	34.96		
id:Familiarity	8.19	2.86		
id:Sound	6.89	2.62		
id	5,103.72	71.44		
Model fit				
Conditional *R*^2^	Marginal *R*^2^			
0.90	0.20			

We conducted a post-hoc analysis for the main effect of the Stroop condition. The response time for the Congruent condition (mean = 648.62 ms, *SE* = 14.63) was longer than that for the Control condition (mean = 631.05 ms, *SE* = 14.63) with statistics: *t* = 2.57, *p* = 0.03, *d* = 0.67, and 95% *CI* = [0.14, 1.2]. The response time for the Congruent condition was shorter than for the Incongruent condition (mean = 716.87 ms, *SE* = 14.63) with statistics: *t* = −9.97, *p* < 0.01, *d* = −2.62, and 95% *CI* = [−3.31, −1.91]. Moreover, the response time for the Control condition was shorter than the Incongruent condition: *t* = −12.54, *p* < 0.01, *d* = −3.29, and 95% *CI* = [−4.08, −2.5]. We conducted a post-hoc analysis for the effect of familiarity. The response time in the Familiar condition (mean = 668.66 ms, *SE* = 14.14) was longer than in the Unfamiliar condition (mean = 662.37 ms, *SE* = 14.14) with statistics: *t* = −2.57, *p* = 0.02, *d* = −0.95, and 95% *CI* = [−1.71, −0.18]. *Post-hoc* analyses were conducted on the significant interaction between Sound and Time. Although the effect of Time was not significant in both the crying sound and white noise conditions, there was a decrease in response time from Time 1 (mean = 673.26 ms, *SE* = 14.94) to Time 2 (mean = 657.3 ms, *SE* = 14.94) in the crying sound condition (*t* = 1.66, *p* = 0.11, *d* = 0.58, and 95% *CI* = [−0.12, 1.27]). In the white noise condition, the response times were as follows: Time 1 (mean = 668.36 ms, *SE* = 14.94) and Time 2 (mean = 663.14 ms, *SE* = 14.94) with statistics *t* = 0.54, *p* = 0.59, *d* = 0.19, and 95% *CI* = [−0.5, 0.87].

### Error rate

Similarly, for the error rate, we applied a LMM. Due to some variance components being zero or extremely small, the random effects for id:Sound and id:Time were excluded from the model. After comparing the fit of each model (Table 2), Model 3, which only included main effects, was chosen as the final model (refer to Table 4). Within this model, there was a significant main effect for Condition. The error rate in the Incongruent condition (mean = 0.018, *SE* = 0.005) was higher than in both the Control (mean = 0.001, *SE* = 0.005) and Congruent conditions (mean = 0.002, *SE* = 0.005) with statistics Control-Incongruent: *t* = −2.58, *p* = 0.03, *d* = −0.68, and 95% *CI* = [−1.2, −0.14] and Congruent-Incongruent: *t* = −2.69, *p* = 0.02, *d* = −0.71, and 95% *CI* = [−1.23, −0.17]. No significant difference was observed between Control and Congruent (*t* = −0.11, *p* = 0.99, *d* = −0.03, and 95% *CI* = [−0.54, 0.49]). Furthermore, the main effect of Time Point was significant, indicating that the error rate was higher at Time 2 (mean = 0.009, *SE* = 0.003) than Time 1 (mean = 0.006, *SE* = 0.003) with statistics *t* = −2.05, *p* = 0.04, *d* = −0.17, and 95% *CI* = [−0.33, −0.01].

**Table 4:** Linear mixed model results for the error rate.

Fixed effects				
	F	Df	Df.res	*p* value
Age	2.48	1	26	0.13
Child age	0.52	1	26	0.48
Interval	1.3	1	26	0.26
Condition	4.63	2	58	0.01
Sound	0.62	1	598	0.43
Familiarity	0.08	1	29	0.77
Time	4.21	1	598	0.04
Random effects				
	Variance	SD		
id:Condition	0.001	0.02		
id:Familiarity	0.0001	0.01		
id	0.0001	0.01		
Model fit				
Conditional *R*^2^	Marginal *R*^2^			

### Association of attentional biases and with subjective ratings for crying and postpartum depression symptoms

The correlation coefficients between response time for each condition and EPDS scores were calculated at each time point (Supplementary Table S2). Overall, there was a positive correlation. Notably, at Time 2, a significant correlation was found between the response time under the incongruent condition of the Stroop task, where color and text do not match, and the EPDS scores. This suggests that mothers with higher postpartum depression (PPD) symptoms performed worse in conditions of high task difficulty.

**Figure 2:**
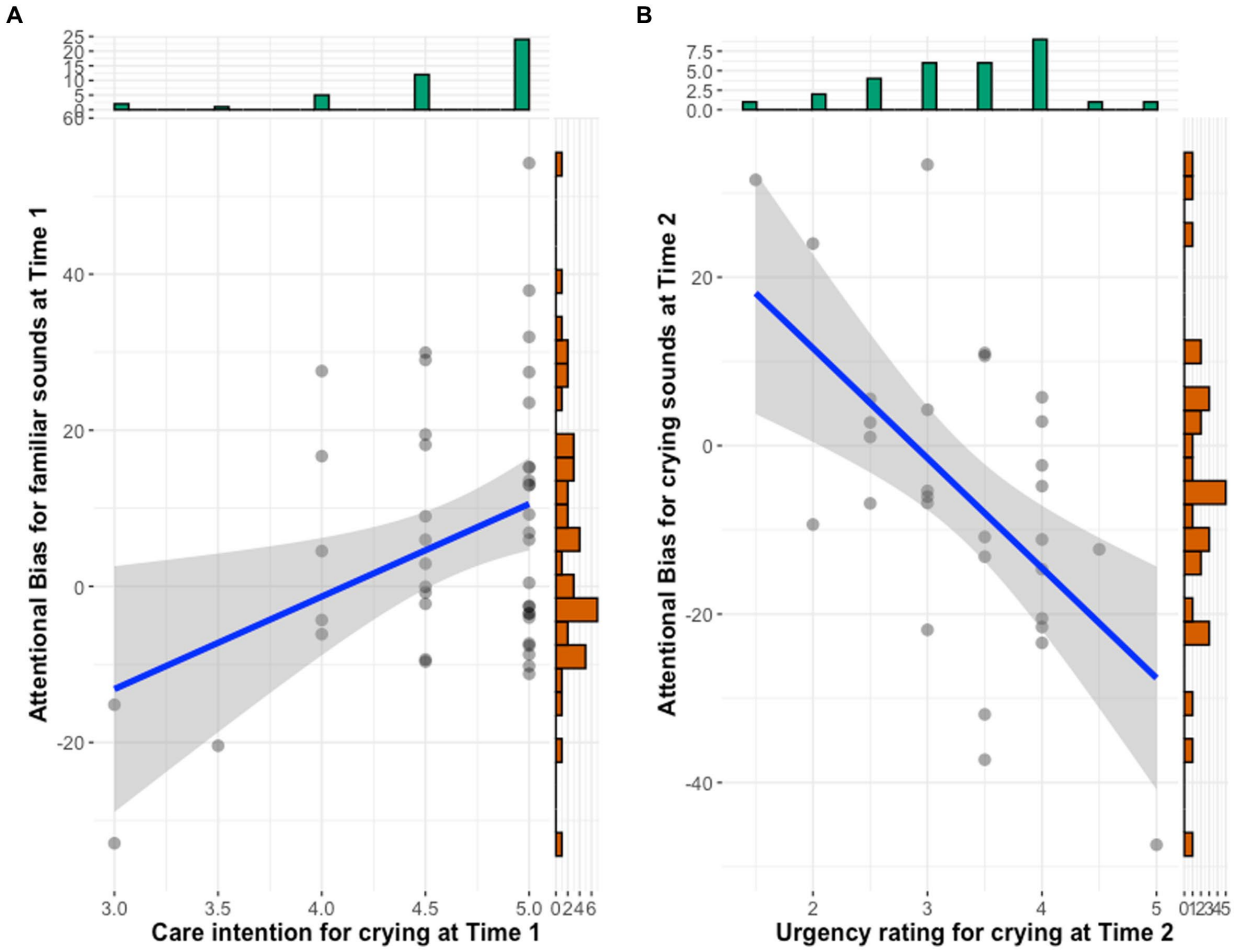
Scatter plots between attentional bias and subjective ratings. **(A)** Bias toward familiar infant vocalization and caregiving intention toward crying at Time 1. **(B)** Bias toward infant cry sound and urgency toward crying at Time 2.

To examine the relationship between attentional bias toward crying and own infant’s vocalizations and subjective ratings and maternal depressive symptoms, we conducted correlation analyses of the relationships among variables at each time point (Table 5). The results showed that greater bias toward one’s own infant’s vocalizations was significantly and positively correlated with intention to care for infant crying at Time 1 (*r* = 0.37, *p* = 0.013). Mothers whose response time was slower when they heard their own infant’s cry than when they heard unfamiliar infant’s cry rated their caregiving intentions toward crying higher (Figure 2A). This relationship was no longer significant at Time 2. At Time 2, a higher bias toward crying was significantly negatively correlated with urgency toward crying (*r* = −0.58, *p* = 0.001). Mothers whose response times were longer when they heard infant crying than when they heard white noise rated the urgency of crying as lower (Figure 2B).

**Table 5:** Correlation matrix of attentional bias and subjective ratings of crying at each time point.

Time	Bias	Time 1						Time 2					
		Urgent	Arousal	Healthy	Valence	Care	EPDS	Urgent	Arousal	Healthy	Valence	Care	EPDS
Time 1	Own	0.13	0.22	−0.24	−0.01	**0.37**	0.25						
	Cry	0.18	0.02	−0.07	0.15	−0.12	0.10						
Time 2	Own							−0.07	0.07	0.00	−0.18	0.18	0.23
	Cry							**−0.57**	−0.33	0.09	0.01	−0.13	−0.03

Boldface indicates statistical significance (*p* < 0.05).

## Discussion

This study investigated the existence of attentional bias toward infant crying and its longitudinal changes through the postpartum period for new mothers. The results indicated the presence of attentional bias toward the sounds of their own infants. Such attentional bias was positively correlated with caregiving intentions at Time 1. The changes in reaction time from Time1 to Time2 in the crying and white noise conditions were significantly different, with the decrease being more pronounced in the crying condition. Furthermore, at Time 2, higher attentional bias toward infant crying was associated with less urgency in responding to the crying. This study represents the first exploration of longitudinal changes in caregivers’ attentional bias toward infant crying.

The perinatal period is characterized by dramatic neurobiological and cognitive changes that enhance sensitivity to infant cues, promoting infant survival and the formation of mother–infant bonds (Barrett and Fleming, 2011; Hoekzema et al., 2017, 2020; Numan, 2020). Concurrently, this period also carries an increased risk for mental health disorders such as postpartum blues, PPD, and disorders of mother–infant bonding (Workman et al., 2012). The interaction between increased sensitivity and mental health risk may involve a balance of systems; while mothers are required to respond sensitively to infant cues, they must also manage and regulate these responses through emotional regulation and executive functions to display well-regulated caregiving behaviors (Rutherford et al., 2015; Kim et al., 2016; Swain and Ho, 2017). Further, while empathy for a crying infant is a crucial mental function motivating caregiving behavior (Hipwell et al., 2015; Swain and Ho, 2017), empathy is not automatically invoked and can decrease in situations in which cognitive resources are constrained, subsequently lowering caregiving intentions (Hiraoka and Nomura, 2016). This failure to regulate may increase the risk of inappropriate caregiving and worsening of mental health (Crouch et al., 2018, 2021). Both perinatal depression and anxiety have been associated with alterations in the cognitive and neurobiological processing of infant crying (Ho and Swain, 2017; Guo et al., 2018), suggesting a bidirectional relationship between responsiveness to infant crying and worsening mental health.

In this study, we found no significant relationship between bias toward infant crying and depressive symptoms. However, especially at Time 2, mothers with higher depressive symptoms displayed shorter response times in those conditions with higher task difficulty. This is consistent with the previous findings that depressive symptoms do not inhibit cognitive task performance across the board, but may cause a decline in performance in more challenging tasks (Hammar et al., 2010). However, in most individuals, disturbances in postpartum mental health gradually decrease naturally over time (Yu et al., 2020). Additionally, any cognitive decline observed during the perinatal period tends to be temporary, with recovery noted around the time of weaning (Workman et al., 2012; Orchard et al., 2023). It has been suggested that the immediate postpartum state of preoccupation with infant stimuli is unique and requires gradual recovery from it (Winnicott, 1956). This implies that the gradual decrease in the sensitivity and recovery of cognitive functions through the postpartum period can improve the regulation of psychological and behavioral responses to infant cues, thereby attenuating parenting stress and risk of worsening mental health.

The structural changes in the brain, with volume reductions in multiple regions from pregnancy to the postpartum period (Hoekzema et al., 2017), also recover postpartum, suggesting a return to the pre-pregnancy state (Kim et al., 2010; Luders et al., 2020). Both other-oriented and self-oriented types of empathy toward infant crying exist and it has been shown that parent-oriented beliefs increase compared with empathy oriented toward infant emotions (Hiraoka et al., 2021). The decrease in bias observed in our study may reflect the early stages of these adaptive processes, including improved cognitive function and reduced bias toward infant stimuli. Future work could adopt comprehensive measures of cognitive function as well as physiological and neurobiological responsiveness to infant crying to explore the adaptive process and mechanisms of responding to infant crying more in depth.

The results of our study suggest that there is a reduction in response times to crying in general, irrespective of whether the cry is from one’s own infant or an unfamiliar infant. This indicates that the adaptation might not be specific to one’s own infant’s cry due to experience but rather a general adaptation to infant crying. The development of a mother-infant bond is proposed to follow a model that involves a recognition process and a persistent attraction process (Numan and Young, 2016). Depending on the species, there might be a biological predisposition to selectively attend to one’s own offspring or to attend non-selectively to any juvenile of the same species. Especially in species where females give birth to altricial offspring, there is often non-selective recognition at the time of birth. This means that general juvenile stimuli can access the maternal motivation system in the brain, leading to caregiving behaviors directed at any juvenile of the same species. Whether human parents show a specific response to their infant’s cry is still under debate (Li et al., 2018; Witteman et al., 2019). Witteman et al. (2019) showed through a meta-analysis that there are differences in processing infant stimuli based on parental status, and their studies included cries from unfamiliar infants. This suggests the possibility that brain processing of infant cries in general might change based on parental status or caregiving experience. From this perspective, it is conceivable that caregiving experience might alter responses not only to one’s own infant’s cry but also to the cries of unfamiliar infants. However, the lack of a significant effect does not necessarily mean there is no difference, so drawing firm conclusions is challenging. Rilling (2013) has suggested a classification for the processing of infant cries into five stages: auditory processing, the alarm signal response, the reward system, emotional empathy, and finally, cognitive empathy. The attentional bias to crying measured in this study might belong to the earlier stages, indicating a very automatic and instinctual response. If variables from later stages were measured, there might be an interaction between familiarity and time point. In the future, exploring the effects of familiarity on changes from perspectives other than attentional bias will be meaningful.

The increase in correct response rates at Time 2 might indicate that the participants had adapted to their infants’ cries, leading to automatic responses and resulting in less attentive responses, rather than a simple decrease in response times. Therefore, the results should be interpreted as a balance between improved processing speed and accuracy, reflecting the natural course of adaptation to the role of caregiving.

At Time 2, mothers who rated crying as more urgent were less biased toward crying and responded faster due to crying. Previous studies have shown that hearing infant crying improves motor task performance (Parsons et al., 2012; Young et al., 2015). Initially, mothers do not understand the reason for the crying and use a trial-and-error approach to sooth crying infants (Drummond et al., 1993; Kurth et al., 2014). However, with experience, mothers begin to understand the reasons for infant crying and adopt methods to cope with it (Drummond et al., 1993; Kurth et al., 2014). At Time 1, while mothers may have responded uniformly regardless of their level of urgency, urgency and response time became linked as they gained experience. At Time 2, task performance due to crying may have increased among those participants who rated their level of urgency higher.

Response times were longer in the trials in which their own infants’ cries were presented, indicating a bias toward their own infant’s cry. The envelope was retained when converted to white noise, which may have led to specific cognitive processing based on the envelope properties of the infant’s cry, even in the white noise condition. Primates, including humans, are semi-mature at birth, and mothers form selective attachments to their own children to prevent them from being confused with other young animals in the group and becoming unable to care for them (Numan, 2020). Areas involved in reward and value judgments, such as the orbitofrontal cortex and anterior insula, are more activated by their own infant’s cry than by the cries of unfamiliar infants (Noriuchi et al., 2008). Doi and Shinohara (2012) found that their own infant’s cry enhances the P300 component, which reflects saliency, compared with unfamiliar infants’ cries. The cry of one’s infant can thus be interpreted as a more salient and attention-grabbing stimulus. At Time 1, we found that the higher bias toward their own infant’s cry, the higher the mother’s caregiving intentions. This result is in line with the lower bias toward infant face stimuli among mothers with perinatal depression and bonding difficulties (Pearson et al., 2010, 2011). Unlike general attentional bias toward infant crying, however, there was no significant decrease in attentional bias at Time 2. A mother’s feelings toward her own infant are formed during pregnancy and in its first year of life (Kinsey and Hupcey, 2013). Bias toward one’s own infant may have held at Time 2, as an eight-month-old infant who is not yet able to move satisfactorily on their own may require selective attention and care.

A major strength of this study is that we were able to obtain basic cognitive data from a homogeneous sample by controlling for the infant’s age in months and mother’s parenting experience as much as possible. However, this study has certain limitations. We only employed crying as a stimulus and did not use other infant sounds. In addition to crying, other means of infant communication include laughter and babbling (Bowlby, 1969). While crying serves as a crucial and immediate call for parental attention and can notably contribute to parental stress, infants communicate through a diverse array of vocalizations. This research was a preliminary attempt to measure changes in cognitive biases towards infant vocalizations. To ensure clarity and avoid an overly intricate experimental design, we limited our study to the sound of infant crying. Furthermore, compared to crying, which is present from birth, vocalizations like babbling and laughter emerge later in development (Bowlby, 1969; Sroufe, 2005; Pearson et al., 2013). Given the early stage of our assessment (approximately 2 months postpartum), we were concerned that not all participants would have experienced these sounds with their infants, further justifying our focus on crying. Future studies that encompass a broader spectrum of infant vocalizations could offer a more comprehensive understanding of maternal attention and its developmental trajectory. Additionally, our study scrutinized changes at two distinct postpartum time points, specifically two and 8 months. However, integrating additional time points might have led to divergent outcomes. Notably, the frequency of infant crying reaches its zenith during the initial one or 2 months and then sees a gradual decline (Barr, 1990; Vermillet et al., 2022). This pattern suggests that our study might capture a mother’s gradual habituation from the peak crying period. Yet, the cognitive bias could be more pronounced and perhaps even intensify when juxtaposing the immediate postpartum phase (a mere one or 2 days after childbirth) with the subsequent one or 2 months. The trajectory of these changes could potentially deviate from a linear pattern. Supporting this, Bilgin and Wolke (2020) discerned evolving patterns in maternal reactions to infant cries. They reported that the fraction of mothers seldom letting their infants cry unabated reduced from 0 to 3 months but stabilized from 3 to 18 months. In contrast, mothers frequently permitting their infants to cry saw little alteration from 0 to 6 months but experienced a surge from 6 to 18 months. Moreover, certain factors might modulate the trajectory of these changes. For instance, Hiraoka et al. (2021) identified varied paths in caregivers’ perceptions regarding infant crying, partly influenced by the infant’s inherent temperament, specifically surgency. Neural responses to infant crying can change depending on the mode of delivery and breastfeeding (Swain et al., 2008; Kim et al., 2011), and postnatal interventions have been shown to reduce parenting stress, leading to changes in brain functional connectivity (Swain et al., 2017). In summation, the collective evidence underscores that maternal reactions to infant cries do not follow a uniform trajectory and may occasionally deviate from a linear pattern. Future research endeavors would benefit immensely from a comprehensive longitudinal approach, encompassing diverse time points, varied determinants, and a more expansive participant demographic. While this study employed the Stroop task, which reflects inhibitory processes among various cognitive functions, the results may vary, if different tasks are used. Various cognitive functions are required to process an infant’s cry (Kim et al., 2016; Swain and Ho, 2017). For instance, an infant’s cry can also inhibit performance in N-back tasks that reflect working memory (Hechler et al., 2015). Future research, by implementing multiple tasks measuring various cognitive functions, could provide a deeper understanding of which functions are inhibited or, in other words, which functions are necessary to process an infant’s cry. Lastly, this study did not fully control for potential confounding factors that might influence the results. Factors like participants’ immediate fatigue, sleep quality and duration, and hormonal levels are known to affect responses to an infant’s crying (e.g., Kurth et al., 2011; Probst et al., 2017; Voorthuis et al., 2019). Measuring and controlling these factors in detail would allow for more robust analyses. Furthermore, considering these factors not only reduces errors but also contributes to uncovering underlying factors associated with caregivers’ responses to crying. In future research, it will be essential to obtain these psychological and physiological metrics and integrate them into the analytical model.

The presented findings provide evidence that attentional bias toward infant crying varies over time and that this bias reduces with parenting experience. One interpretation of this result is that bias toward infant crying reduced as mothers adapted to crying and their processing became more automatic. The adaptive aspect of infants’ bias toward stimuli is also under discussion (Lucion et al., 2017; Endendijk et al., 2018), and the adaptive nature of this change will also require further study. To understand whether it is desirable to maintain or reduce bias toward infant crying or, conversely, what is occurring in individuals who increase it, more samples and time points will be needed. Given that infant crying, while necessary for infant survival, can be a trigger for parenting stress and child abuse, research on the process of adaptation to crying is clinically vital and our study is expected to contribute to this area of research.

## Data availability statement

The datasets presented in this study can be found in online repositories. The names of the repository/repositories and accession number(s) can be found at: https://osf.io/by5me/?view_only=1b3a69f4d7884ed1902ff3afe5e57b55.

## Ethics statement

The studies involving humans were approved by the Research Ethics Committee of University of Fukui. The studies were conducted in accordance with the local legislation and institutional requirements. The participants provided their written informed consent to participate in this study.

## Author contributions

DH conceived and designed the project, analyzed the data and drafted the manuscript. MO, SM, and YY recruited the participants. DH, NS, and KM performed the experiments and collected the data. All authors revised the manuscript, contributed to the article, and approved the submitted version.

## Footnotes

^1^ http://audacity.sourceforge.net
