# Supplementary material for: Longitudinal changes in attention bias to infant crying in primiparous mothers
Source: Front Behav Neurosci. 2023 Sep 22;17:1192275. doi: 10.3389/fnbeh.2023.1192275 (PMC10556249; doi:10.3389/fnbeh.2023.1192275)
Supplement: Supplementary file 1 [file Data_Sheet_1.docx]

# Supplementary Information

Table S1. The reasons or contexts of the recorded infant's cries.

| Reason | Time 1 | Time 2 |
| --- | --- | --- |
| Before/During diaper change | 0 | 1 |
| During car ride | 0 | 1 |
| Following | 0 | 1 |
| Hungry | 22 | 5 |
| Refusing car seat | 0 | 1 |
| Unknown | 6 | 4 |
| Waking up/Before sleep | 2 | 12 |
| Wants attention | 0 | 5 |

Table S2. Correlation Coefficients between EPDS Scores and Response Times for Each Condition at Each Time Point

|  | congruent | | | | control | | | | incongruent | | | |  |
| --- | --- | --- | --- | --- | --- | --- | --- | --- | --- | --- | --- | --- | --- |
|  | noise | | cry | | noise | | cry | | noise | | cry | |  |
|  | unfamiliar | familiar | unfamiliar | familiar | unfamiliar | familiar | unfamiliar | familiar | unfamiliar | familiar | unfamiliar | familiar |  |
| Time 1 | 0.18 | 0.29 | 0.18 | 0.18 | 0.16 | 0.16 | 0.20 | 0.27 | 0.19 | 0.17 | 0.21 | 0.24 |  |
| Time 2 | 0.08 | 0.11 | 0.16 | 0.17 | 0.21 | 0.34 | 0.16 | 0.19 | **0.37** | **0.41** | **0.39** | 0.36 |  |
| Note: Boldface indicates statistical significance (*p* < .05). | | | | | | | | | | | | | |


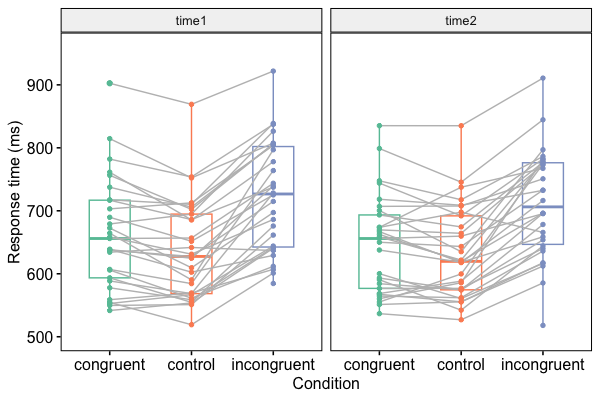


Figure S1. Response time of Stroop condition at each time point. Each box plot displays the median (central line), first and third quartiles (box boundaries), and the maximum and minimum values within 1.5 times the interquartile range (whiskers). Each point represents an individual participant's score.


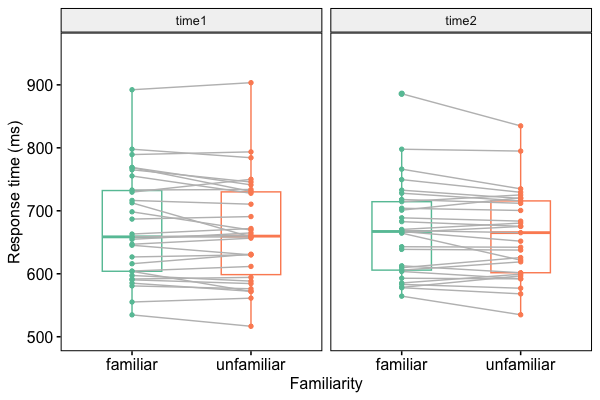


Figure S2. Response time of Familiarity condition at each time point. Each box plot displays the median (central line), first and third quartiles (box boundaries), and the maximum and minimum values within 1.5 times the interquartile range (whiskers). Each point represents an individual participant's score.
